# Real-world management of opioid use disorder in primary care 2015–2019: associations between clinical practice attributes, diagnosis, and treatment

**DOI:** 10.1080/09581596.2026.2676423

**Published:** 2026-05-25

**Authors:** Esther E. Velásquez, Mathew V. Kiang

**Affiliations:** a Center for Population Health Sciences, Stanford University, Stanford, CA, USA; b Department of Epidemiology and Population Health, Stanford University, Stanford, CA, USA

**Keywords:** Opioid use disorder, access to treatment, primary care, epidemiology, health care delivery

## Abstract

Expanding the treatment of opioid use disorder (OUD) remains a national priority and given constraints on treatment access, primary care may be an essential treatment context. While intervention studies within primary care have demonstrated efficacy, barriers to successful implementation and scale-up persist. The purpose of this work is to describe real-world management of OUD in primary care and assess how attributes of clinical practices are associated with rates of diagnosis and treatment. This observational study uses electronic health records from the American Family Cohort, a research dataset derived from PRIME, a Qualified Clinical Data Registry for primary care. The analytic cohort includes 854 clinical practices that were active between 2015-01-01 and 2019-12-31 and 4,767,971 patients attending those clinical practices. Overall, the OUD prevalence was 0.59% between 2015 and 2019. Approximately two-thirds of all patients with OUD and nearly 90% of all visits related to OUD were concentrated in only 50 primary care practices (5.8%). High-prevalence practices tended to be in metropolitan areas and have a higher ratio of physicians and behavioral health providers per patient. The concentrated distribution of patients with OUD in our study suggests that primary care practices are an important but underused tool for OUD. Further, targeted investment in a small number of high-volume practices may be more efficient for population health impact than universal scale-up.

## Introduction

Since 1999, over 1 million people in the United States have died from drug overdoses, primarily driven by opioids (Hedegaard et al., 2020; Spencer, 2024). A primary driver of opioid-related deaths is opioid use disorder (OUD), a condition affecting an estimated 2.5 million US adults in 2021 alone (Jones et al., 2023). Highly effective, safe medications for opioid use disorder (MOUD) have been available in the US for decades (e.g. buprenorphine, extended-release naltrexone, and methadone) (Nasem & Division, 2019). However, despite efforts to improve MOUD access, treatment access remains low, especially among rural residents, youth, pregnant women, and racially and ethnically minoritized groups (National Center for Injury Prevention and Control, 2022). Even when treatment is accessible, treatment initiation is low – only an estimated 11% of those with OUD are actively on treatment (Morgan et al., 2022). Last, among people who initiated treatment, long-term retention is less than 25% (Chua et al., 2023), and discontinuation of treatment is a risk factor for overdose (Larochelle et al., 2022).

Given the pervasiveness of OUD across the United States, the need for treatment of patients with OUD exceeds the capacity of specialty treatment settings (Jones et al., 2015). Expanding OUD treatment efforts in primary care may be a viable solution since such care may be more accessible than treatment in specialty care settings and patients may visit their primary care providers for other health needs, including preventive screenings, immunizations, and prenatal care. Further, there is evidence that primary care settings are more likely to test for HIV and hepatitis C and use regular toxicology testing than behavioral health specialists, which reflects added benefits of this treatment setting (Gertner et al., 2020).

Existing implementation studies have demonstrated efficacy of primary care-based interventions for OUD resulting in increases in both patients diagnosed with OUD as well as patients treated with MOUD (Cheng et al., 2022; Hall et al., 2025). And yet implementation research has also revealed barriers to adoption and sustainability ranging from structural barriers (e.g. inflexibility in scheduling visits) (Ukaoma et al., 2025) to provider barriers (e.g. perceptions of ability to provide adequate care) (Austin et al., 2023) to patient barriers (e.g. not aware MOUD is available in primary care) (Del Pozo et al., 2024). A useful finding from these efforts is that a one-size fits all strategy may be ineffective and inefficient and implementers would benefit from guidance on knowing where to start and where to focus effort (Cheng et al., 2022). Thus, a first step in considering how to nationally address OUD treatment in primary care is understanding the current status of OUD treatment in primary care from a population health perspective. Yet much existing evidence from implementation and epidemiologic studies is often limited due to small sample size, limited geographic scope, a focus on patient-level characteristics irrespective of treatment setting, or a focus on provider-level characteristics irrespective of treatment setting –which may limit generalizability and have minimal applicability for informing management of OUD in primary care (DeFlavio et al., 2015; Duncan et al., 2020; Ober et al., 2022; Walley et al., 2008).

The purpose of this work is to describe the real-world management of OUD in the primary care context by integrating information on patient, provider, and clinic characteristics. We leverage a novel dataset consisting of 4.7 million primary care patients seen in 854 clinical practices across the United States. In this descriptive analysis we describe the attributes of the clinical practices including the composition of the clinical care team, the proximity of the clinical practice to an urban center, OUD diagnosis prevalence, and proportion of patients prescribed MOUD. Here we emphasize the variation across clinical practices in the diagnosis and treatment of OUD and the relationship of these outcomes with attributes of the clinical practices. Further, we consider how the demographic distribution of the patients with OUD in the primary care context compares with other national estimates to consider how well different subgroups are captured in primary care.

## Methods

Our data come from the American Family Cohort (AFC), a research dataset of electronic health records derived from PRIME, a Qualified Clinical Data Registry established by the American Board of Family Medicine (Stanford Center for Population Health Sciences, n.d). PRIME is the largest national registry for primary care and family medicine and generates quality performance metrics for participating providers and clinical practices. The AFC is a unique dataset because it includes self-reported patient sociodemographic data including gender, race, and marital status; covers patients with both public and private insurance and patients and clinical practices in small towns and rural areas; and offers granular geospatial data on both patient home residence and clinical practice locations, which enable us to assess area-level outcomes such as area-level social deprivation. Data for the current study is available for approved research purposes through the American Family Cohort Research Consortium, and researchers can apply for direct access through the Consortium (americanfamilycohort.org). For this analysis, we used the AFC OMOP RIF (Version 5.0; DOI:10.71778/V2DW-7A53).

For this observational study, clinical practices were eligible for inclusion in the study if they remained active (i.e., contributed health encounter data) for the entire period between 2015-01-01 and 2019-12-31. AFC is an open cohort and at the time of this research, the AFC data were available through 2023. We assessed all 3 to 5-year periods between 2015 and 2023 and chose the 2015–2019 period to maximize the number of clinical practices that were observed continuously. Of 1,321 clinical practices observed during the study period, 854 (64.6%) met the inclusion criterion. There were 6,422,789 unique patients with recorded encounters during the study period; of those patients, 4,767,971 (74.2%) attended one of the selected clinical practices.

During the study period, we identified OUD by a diagnosis code for opioid use, abuse, or dependence in the electronic health record corresponding to diagnosis codes from the International Classification of Diseases, Clinical Modification; we used both the Ninth Revision and the Tenth Revision (21, 2024). We refer to these diagnosis codes as opioid-related disorders (ORD).

We summarized practice characteristics and compare attributes of clinical practices by the prevalence of ORD within the clinical practices. Using the address of clinical practices, we assigned US Census Region (Midwest, Northeast, West, and South) by state and assigned rural–urban commuting area (RUCA) codes by zip code. Patient panel size was defined as the number of unique patients with a healthcare encounter at the clinical practice during the study period. We calculated the crude prevalence of ORD for the study period as the number of patients with a diagnosis during the study period divided by the total number of patients observed and repeat this process for each clinical practice as well to observe the variation in identification and diagnosis of ORD across the clinical practice sample. We generated confidence intervals for prevalence estimates using the Clopper-Pearson method (Parker et al., 2017). Further, we ranked clinical practices 1st to 854th by the number of unique patients with ORD during the study period such that the clinical practice ranked 1st diagnosed the largest number of patients with ORD. The purpose of this analysis is to assess the distribution of patients with ORD across the sample of clinical practices.

Next, we quantified the relative and absolute frequencies of different types of providers by ORD ranking to identify any relationship between the composition of clinical teams and clinical practice rank. To identify provider type, we extracted the National Provider Identifier (NPI) from the electronic health record and linked it to NPI files hosted by the Centers for Medicare and Medicaid National Plan and Provider Enumeration System (NPPES) (publicly available at https://download.cms.gov/nppes/NPI_Files.html). Then we linked the healthcare provider taxonomy code from the NPI files to the National Uniform Claim Committee (publicly available at https://nucc.org). With these linkages we grouped providers in our data using the NUCC groups: allopathic and osteopathic physicians, nurse practitioners and physician assistants, and behavioral health and social service providers. We considered all other provider groups to be ‘other providers’ and created two additional, mutually exclusive groups: (1) allopathic and osteopathic physicians with a specialization of addiction, anesthesiology, pain, palliative care, or psychiatry were classified as ‘ORD physicians’ and (2) nurse practitioners and physician assistants with the specialization anesthesiology, mental health, or psychiatric were classified as ‘ORD nurse practitioners and physician assistants’.

Finally, we calculated the overall proportion of patients with ORD who were ever prescribed MOUD during the study period. To assess the variation in proportion of patients prescribed MOUD by clinical practice, we restricted to clinical practices with 10 or more patients with an ORD diagnosis to ensure a large enough denominator to produce a meaningful proportion. To identify MOUD in the electronic health record, we used Observational Medical Outcomes Partnership (OMOP) standardized medical concepts for buprenorphine and naltrexone (Supplemental Table 1).

The latter portion of our analysis focused on the patients with an ORD diagnosis with the aim to describe their demographic characteristics as well as their length of engagement with the clinical practice. Patient demographic characteristics including gender, race, Hispanic or Latino ethnicity, marital status, and date of birth were extracted from the electronic health record. Age was calculated as the first documented date of an OUD diagnosis during the study period. Because of the high prevalence of missing values for race (29.8%) and Hispanic or Latino ethnicity (45.5%) (Supplemental Table 2), we imputed race for missing values: details have been previously published (Lu et al., 2024). We assigned (RUCA) codes to patients using the zip code of home residence and Social Deprivation Index values using the census tract of the patient’s home residence. All analyses were descriptive in nature with no statistical significance testing. Data management was performed using Structured Query Language (SQL) within the Redivis secure computational environment, (Redivis, Inc. https://doi.org/10.71778/v2dw-7a53), which provides a HIPAA-compliant infrastructure for sensitive health data. Descriptive statistical analyses and visualizations were generated using R version 4.5.2.

This study was reviewed and approved on January 26, 2024, by the Stanford Institutional Review Board (Protocol IRB-73460) at Stanford University; the need for written informed consent was waived. The researchers confirm that all study activities were conducted in accordance with the Declaration of Helsinki.

## Results

**Figure 1.**
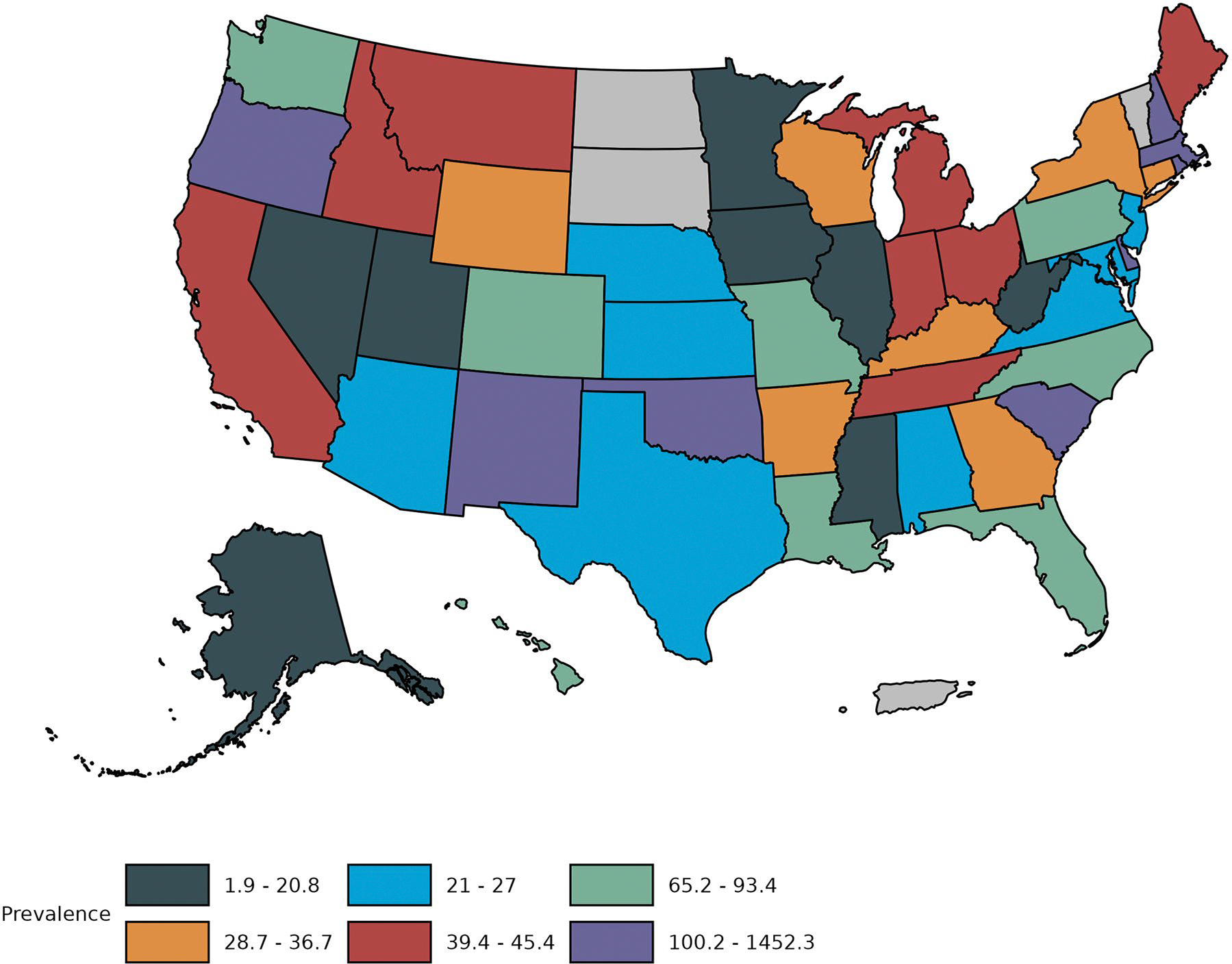
Patients with opioid use disorder per 10,000 primary care patients, 2015–2019.

During the 5-year study period, we continuously observed 854 clinical practices located in 47 states and Washington, D.C. There were 4.7 million patients observed at these clinical practices with a mean patient panel size of 5,583 (σ = 8,818). Figure 1 displays the ubiquity of ORD within primary care nationally as well as the variability in ORD prevalence by state. Between 2015 and 2019, the median prevalence of patients with ORD in primary care was 25.9 per 10,000 patients and ranged from 1.9 in the District of Columbia to 1,452.3 in Delaware. Similarly, we observed substantial variation in the prevalence of ORD by clinical practice: while the overall prevalence of ORD was 0.59% (95% CI 0.58–0.60), 155 clinical practices had 0 patients with ORD. The prevalence of ORD among clinical practices was positively skewed with a median of 0.12% and a mean of 0.92%.

**Figure 2.**
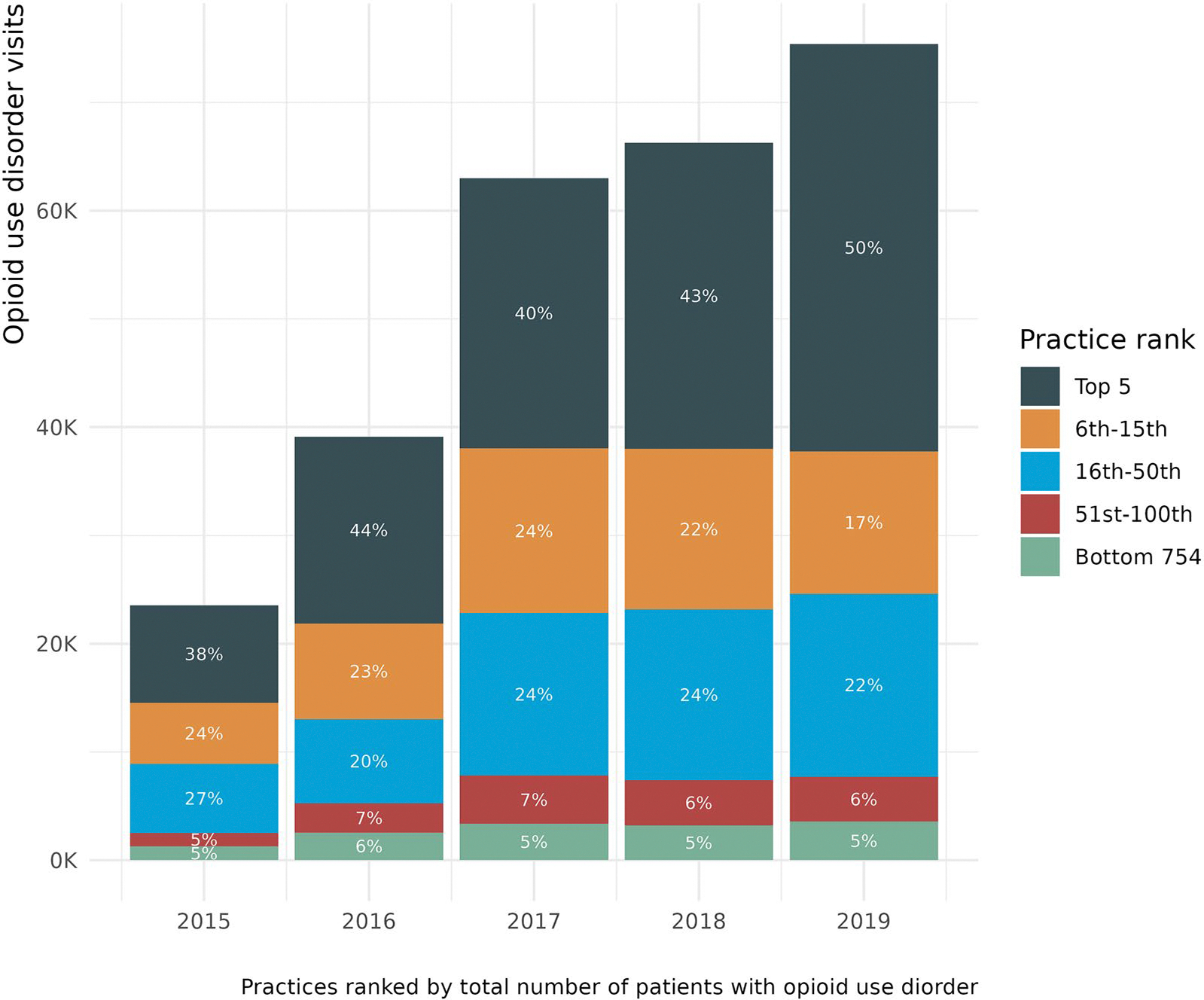
total number of opioid use disorder visits by year, 2015–2019.

A small proportion of practices accounted for most of the patient care for ORD diagnoses. In 2015, the 5 practices with the most patients with ORD accounted for 38.3% of all visits in which ORD was documented (Figure 2). In 2019, the 5 clinical practices with the most patients with ORD accounted for 49.9% of all such visits. For the entire study period, the 50 top ranking clinical practices in terms of patients seen with ORD accounted for 65.6% of all patients with ORD and 86.6% of all visits with ORD. Practice rank was associated with geographical space. Four of the five clinical practices ranked 1st to 5th were located in a metropolitan area and 82% of the top 50 clinical practices were located in metropolitan areas while only 4 of the 34 clinical practices in rural areas were ranked less than 101st (Supplemental Table 3). Practice rank was also associated with composition of the clinical team.

**Figure 3.**
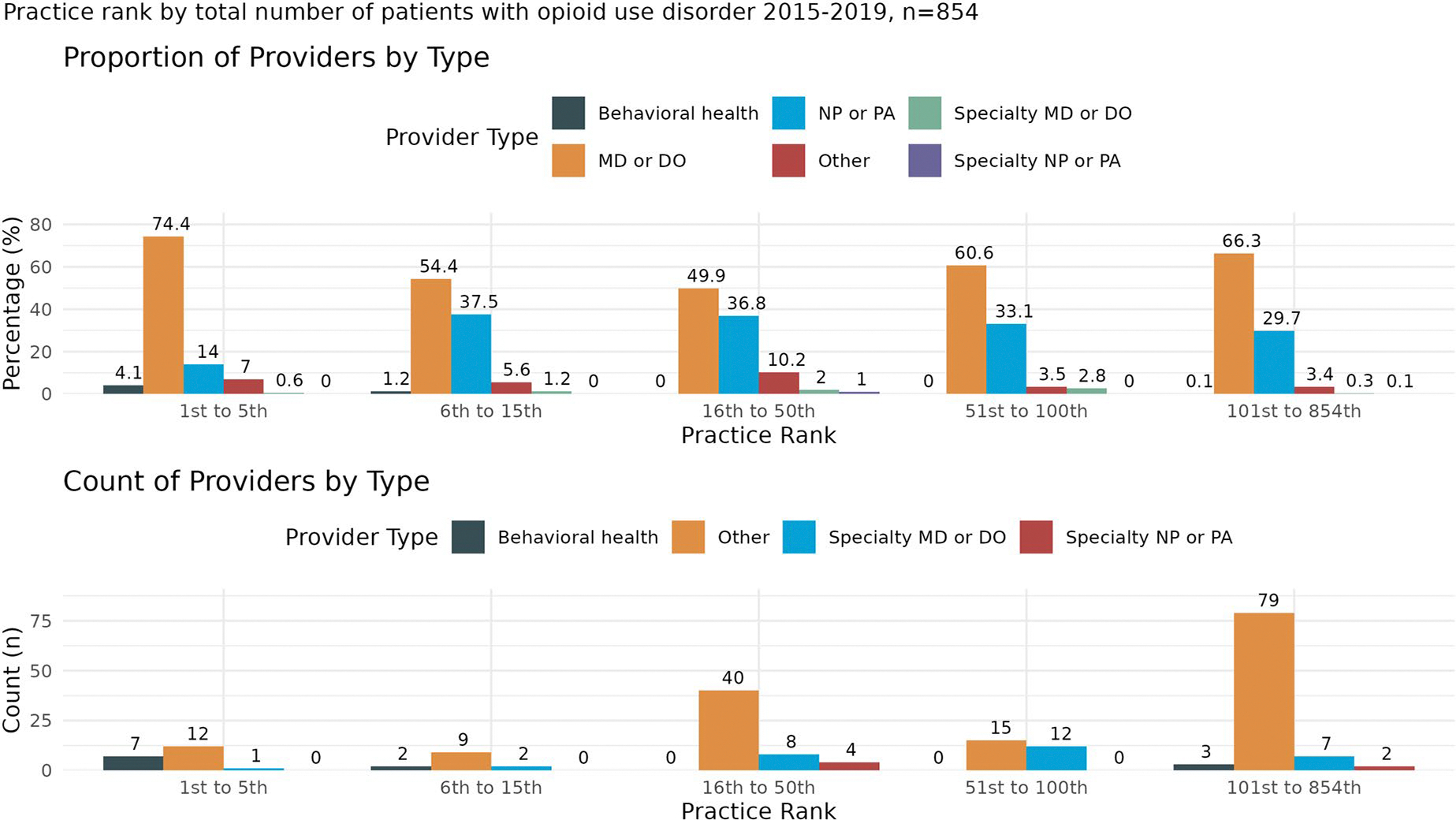
Distribution of providers by type and rank of clinical practice. Practice rank by total number of patients with opioid use disorder 2015–2019, *n* = 854.

The top 5 practices had the highest proportion of physicians (74.4%); practices ranked 101st to 854th had the second highest proportion of physicians (66.3%) among the clinical team (Figure 3). There were 12 behavioral health and social service providers observed across all 854 clinical practices; 7 were at the top 5 practices while 2 were at clinical practices ranked 6th to 15th. We observed 30 physicians with an ORD-related specialty; there was roughly 1 of these providers per every 5 clinics across each ranking group except for clinics ranked 101st to 854th which had ~ 0.05 specialty physicians per every 5 clinics.

Among the 699 clinical practices that had ≥1 patient with ORD, 355 (50.7%) ever prescribed MOUD during the study period to patients with ORD while 301 (43.1%) clinical practices prescribed MOUD to ≥10 patients during the study period. The overall proportion of MOUD among patients with ORD was 33.4% (95% CI 32.9–34.0). The distribution of MOUD proportions among clinical practices with ≥10 patients with MOUD was positively skewed (median = 8.6%; mean = 19.1%). Patient demographic characteristics by MOUD status are reported in Table 1. Overall, 46.8% of patients with ORD were < 45 years of age at first diagnosis during the study period. The most prevalent racial groups among patients with ORD were Black, Hispanic, and White (5.9%, 7.3%, 78.4% respectively). More than 75% of patients resided in the South or West regions of the United States and 13% resided in small towns or rural areas. There were notable differences between patients prescribed MOUD and those not prescribed MOUD. Patients prescribed MOUD were younger on average (mean = 39.8 years) compared to patients not prescribed (mean = 51.9 years). They were also more likely to be White (85.2% versus 75.0%) and less likely to be Black (4.3% versus 6.8%) or Hispanic (3.3% versus 9.3%). Further, patients prescribed MOUD were less likely to live in small towns and rural areas (8.6% versus 15.2%) and more likely to live in areas of less social deprivation. Overall, longitudinal engagement was high among patients with ORD. Of those with an encounter in 2015, 67% had an encounter again in 2019 (Supplemental Figure 1). Of those with an encounter in 2018, 96% had an encounter again in 2019.

## Discussion

In this observational study of 854 primary care practices and 4.7 million patients, we observed 28,168 patients with a documented diagnosis of ORD between 2015 and 2019. While the overall prevalence of ORD was 0.59% (95% CI 0.58–0.60) for the entire study period, there was substantial variation between practices. Similarly, evidence of MOUD prescription varied; overall 33.4% (95% CI 32.9–34.0) of patients diagnosed with ORD were prescribed MOUD; however, at the practice-level the median MOUD proportion was 8.6%. Finally, the 50 clinics with the highest number of patients with ORD diagnosis during the study period accounted for 65.6% of all patients with ORD and 86.6% of all visits with ORD indicated a concentrated distribution of patients with ORD across the clinical practices.

There are several limitations to the current study. First, regarding data source constraints, the AFC captures only patients who accessed primary care during the study period, and case identification depended on provider diagnosis and ICD coding in the electronic health record. Known patient and provider barriers to assessment and disclosure of drug use mean that some patients with OUD were likely misclassified (McNeely et al., 2018; Oni et al., 2019; Palmer et al., 2019), and we did not have data on provider behaviors such as routine screening or whether MOUD was offered and declined. We also lacked data on health insurance status, MOUD dispense counts and refills, and clinical notes. Second, because the analysis is descriptive and does not adjust for patient- or practice-level covariates, the associations we report between practice attributes (team composition, urbanicity, region) and OUD prevalence and MOUD prescribing should not be interpreted causally. Third, regarding generalizability, the AFC is derived from PRIME, a Qualified Clinical Data Registry, and participating practices are not a random sample of US primary care; results describe PRIME-participating practices and may not generalize to non-participating practices, federally qualified health centers, or specialty addiction settings. That said, the overall OUD prevalence in our cohort was within the range of national self-reported survey estimates for the same period (NSDUH 2015–2019), supporting external validity at the population level.

Our primary care estimates show a slightly lower prevalence of OUD than national survey data for the same period. Estimates of national prevalence of past-year OUD from the National Survey on Drug Use and Health (NSDUH) range from 0.9% in 2015 to 0.62% in 2019, with 1.6 million Americans experiencing past-year OUD in 2019 (Substance Abuse and Mental Health Services Administration, 2021). This comparison also highlights subpopulations currently being captured in primary care and for whom treatment gains could be made. Per the NSDUH 2019 results, 55.5% of individuals who may need OUD treatments were male, and women were less likely than men to report past-year MOUD (aRRR, 0.52; 95% CI, 0.29–0.95) (Mauro et al., 2022). In the AFC cohort, 49.0% of patients were male. Following the NSDUH 2019 findings, an estimated 55.1% of people who may have needed OUD treatment were aged 35 years or older; past-year MOUD was not reported among any adolescents (12–17 years) and was reported by only 13.2% of adults ≥50 year (Mauro et al., 2022). AFC patients were predominantly ≥35 years (72.6%; n = 20,449), while only 0.1% were 12–17 years old, and 45.1% were ≥50 years old.

In the current study, the prevalence of OUD compared to national survey data using self-report differed for some racial groups. In the AFC cohort, 0.8% of patients were Asian or Pacific Islander patients and 5.9% of patients were Black; in comparison 2.7% of Asian NSDUH respondents reported OUD, 0.2% Native Hawaiian or Pacific Islander respondents, and 9.9% Black respondents (Mauro et al., 2022). This finding could be driven by reduced healthcare access and/or reduced screening/diagnosis among these subgroups. Evidence from a national Medicare sample suggests that racial disparities in receipt of MOUD persisted despite high levels of healthcare utilization, suggesting that access to care is insufficient to explain the racial inequities (Barnett et al., 2023).

A comprehensive review of existing evidence for implementation models focused on treating OUD within primary care suggested four primary components: (1) MOUD; (2) provider and community education; (3) coordination of OUD treatment with other medical and psychological needs; and (4) psychosocial services (Chou et al., 2016). In the current study, primary care practices with the highest prevalence of ORD had more behavioral health and social service providers and were concentrated in urban areas (with presumably closer proximity to a larger number of community partners) compared to practices with a lower prevalence. This suggests that the clinical practices in our study with a high prevalence of ORD, may have some of the components of efficacious implementation models. Continuity of care and treatment across the life course are a focus of primary care and family practice, which may enhance the suitability of these settings for OUD care (Haggenjos & Yun, 2022). Yet, there is substantial variation in the identification, diagnosis, and treatment of OUD across primary care practices nationally. Additional research is needed to quantify factors influencing rates of diagnosis and treatment of OUD. Specifically, assessing clinical practice screening rates and patient-reports on screeners as well as using unstructured data from clinical notes to learn whether providers discuss MOUD with patients would be valuable data points to maximize the knowledge gained from implementation studies and ultimately maximize treatment potential within primary care.

These findings have direct implications for OUD treatment policy and primary care operations. Because 50 of 854 practices (5.8%) accounted for 65.6% of patients with ORD and 86.6% of ORD-related visits, the marginal return on universal scale-up of MOUD across all primary care practices is likely to be modest compared with the return on targeted technical assistance, workforce development, and care coordination at high-volume practices. The hub-and-spoke MOUD system implemented in Vermont – in which a small number of regional treatment hubs provide induction, complex-case management, and clinical consultation to a wider network of office-based primary care spokes – offers an existing template that aligns with the concentrated distribution observed in our data and that has been associated with substantial expansion of state-level MOUD capacity (Brooklyn & Sigmon, 2017). At the practice level, high-volume primary care practices in our sample also had a higher ratio of behavioral health and social service providers, a pattern consistent with the integrated care components described in evidence-based OUD primary care models.

## Supplementary Material

Supplemental Table 1

Supplemental Table 2

Supplemental Table 3

Supplemental Figure 1

Supplemental data for this article can be accessed online at https://doi.org/10.1080/09581596.2026.2676423.

## Figures and Tables

**Table 1.** Demographic characteristics of primary care patients with opioid use disorder by status of medication for opioid use disorder prescription (n = 28,168).

	No MOUD (N = 18,753)	Prescribed MOUD (N = 9415)	Total (N = 28,168)

**Age First Diagnosis**			
Mean (SD)	51.9 (16.9)	39.8 (12.4)	47.8 (16.5)
Median [IQR]	53.3 [26.3]	37.3 [18.4]	46.9 [26.2]
**Age First Diagnosis**			
<18yr	116 (0.6%)	18 (0.2%)	134 (0.5%)
18–24yr	888 (4.7%)	829 (8.8%)	1717 (6.1%)
25–34	2690 (14.3%)	3178 (33.8%)	5868 (20.8%)
35–44yr	3017 (16.1%)	2436 (25.9%)	5453 (19.4%)
45–54	3413 (18.2%)	1595 (16.9%)	5008 (17.8%)
55–64	4163 (22.2%)	1062 (11.3%)	5225 (18.5%)
65 + yr	4466 (23.8%)	297 (3.2%)	4763 (16.9%)
**Gender** *			
Female	10017 (53.4%)	4327 (46.0%)	14344 (50.9%)
Male	8718 (46.5%)	5082 (54.0%)	13800 (49.0%)
**Race** *			
American Indian or Alaska Native	155 (0.8%)	73 (0.8%)	228 (0.8%)
Asian or Pacific Islander	155 (0.8%)	39 (0.4%)	194 (0.7%)
Black	1266 (6.8%)	402 (4.3%)	1668 (5.9%)
Hispanic	1747 (9.3%)	315 (3.3%)	2062 (7.3%)
White	14,068 (75.0%)	8023 (85.2%)	22,091 (78.4%)
Unknown race	1347 (7.2%)	556 (5.9%)	1903 (6.8%)
**Marital status** *			
Divorced or separated	1750 (9.3%)	580 (6.2%)	2330 (8.3%)
Married	6100 (32.5%)	2110 (22.4%)	8210 (29.1%)
Other or unknown	5645 (30.1%)	4260 (45.3%)	9910 (35.2%)
Single	4320 (23.0%)	2350 (25.0%)	6670 (23.7%)
Widowed	910 (4.8%)	110 (1.2%)	1020 (3.6%)
**US Census region, patient residence**			
Midwest	2351 (12.5%)	836 (8.9%)	3187 (11.3%)
Northeast	1696 (9.0%)	1717 (18.2%)	3413 (12.1%)
South	9830 (52.4%)	5767 (61.3%)	15,597 (55.4%)
West	4743 (25.3%)	1064 (11.3%)	5807 (20.6%)
Unknown	133 (0.7%)	31 (0.3%)	164 (0.6%)
**Rural Urban Commuting Area, patient zip code**			
Metropolitan area	12,618 (67.3%)	7483 (79.5%)	20,101 (71.4%)
Micropolitian area	3137 (16.7%)	1091 (11.6%)	4228 (15.0%)
Small town	1917 (10.2%)	506 (5.4%)	2423 (8.6%)
Rural area	946 (5.0%)	304 (3.2%)	1250 (4.4%)
Unknown RUCA	135 (0.7%)	31 (0.3%)	166 (0.6%)
**Social Vulnerability Index, patient census tract**			
Mean (SD)	0.535 (0.260)	0.494 (0.263)	0.521 (0.262)
Median [IQR]	0.547 [0.411]	0.495 [0.407]	0.530 [0.411]
Unknown census tract	3646 (19.4%)	1710 (18.2%)	5356 (19.0%)

* Three rows were removed from this table due to cell sizes <16. For each of these rows, the total is ≤25 patients. The following rows are masked: other or unknown gender, racial group ‘Multiple races’, and the martial status ‘Partner’.

## Data Availability

Data for the current study is available for approved research purposes through the American Family Cohort Research Consortium, and researchers can apply for direct access through the Consortium (americanfamilycohort.org). Researchers with approved access to the data may also review the analytic cohort, annotation, and code for the current study upon request to the authors.
