# Supplementary material for: Real-world management of opioid use disorder in primary care 2015–2019: associations between clinical practice attributes, diagnosis, and treatment
Source: Crit Public Health. Author manuscript; Available in PMC 2026 Jul 11. (PMC13354046; doi:10.1080/09581596.2026.2676423)
Supplement: Supplemental Table 1 [file NIHMS2181851-supplement-Supplemental_Table_1.docx]

Supplemental Table 1. Observational Medical Outcomes Partnership (OMOP) Common Data Model concept IDs and concept names for buprenorphine and naltrexone

| **Concept ID** | **Concept name** |
| --- | --- |
| 793482 | 0.5 ML buprenorphine 200 MG/ML Prefilled Syringe [Sublocade] |
| 793570 | 1.5 ML buprenorphine 200 MG/ML Prefilled Syringe [Sublocade] |
| 45774486 | 12 HR bupropion hydrochloride 90 MG / naltrexone hydrochloride 8 MG Extended Release Oral Tablet |
| 45774490 | 12 HR bupropion hydrochloride 90 MG / naltrexone hydrochloride 8 MG Extended Release Oral Tablet [Contrave] |
| 40166393 | Abuse-Deterrent morphine sulfate 100 MG / naltrexone hydrochloride 4 MG Extended Release Oral Capsule [Embeda] |
| 40166395 | Abuse-Deterrent morphine sulfate 20 MG / naltrexone hydrochloride 0.8 MG Extended Release Oral Capsule |
| 40166396 | Abuse-Deterrent morphine sulfate 20 MG / naltrexone hydrochloride 0.8 MG Extended Release Oral Capsule [Embeda] |
| 40166398 | Abuse-Deterrent morphine sulfate 30 MG / naltrexone hydrochloride 1.2 MG Extended Release Oral Capsule |
| 40166399 | Abuse-Deterrent morphine sulfate 30 MG / naltrexone hydrochloride 1.2 MG Extended Release Oral Capsule [Embeda] |
| 40166400 | Abuse-Deterrent morphine sulfate 50 MG / naltrexone hydrochloride 2 MG Extended Release Oral Capsule |
| 40166401 | Abuse-Deterrent morphine sulfate 50 MG / naltrexone hydrochloride 2 MG Extended Release Oral Capsule [Embeda] |
| 40166403 | Abuse-Deterrent morphine sulfate 60 MG / naltrexone hydrochloride 2.4 MG Extended Release Oral Capsule |
| 40166404 | Abuse-Deterrent morphine sulfate 60 MG / naltrexone hydrochloride 2.4 MG Extended Release Oral Capsule [Embeda] |
| 40166407 | Abuse-Deterrent morphine sulfate 80 MG / naltrexone hydrochloride 3.2 MG Extended Release Oral Capsule |
| 40166408 | Abuse-Deterrent morphine sulfate 80 MG / naltrexone hydrochloride 3.2 MG Extended Release Oral Capsule [Embeda] |
| 45776270 | buprenorphine / naloxone Buccal Film |
| 1593218 | buprenorphine 0.7 MG / naloxone 0.18 MG Sublingual Tablet |
| 1593220 | buprenorphine 0.7 MG / naloxone 0.18 MG Sublingual Tablet [Zubsolv] |
| 43532942 | buprenorphine 1.4 MG / naloxone 0.36 MG Sublingual Tablet |
| 43532943 | buprenorphine 1.4 MG / naloxone 0.36 MG Sublingual Tablet [Zubsolv] |
| 45892567 | buprenorphine 11.4 MG / naloxone 2.9 MG Sublingual Tablet |
| 45892569 | buprenorphine 11.4 MG / naloxone 2.9 MG Sublingual Tablet [Zubsolv] |
| 42898499 | buprenorphine 12 MG / naloxone 3 MG Sublingual Film |
| 42898500 | buprenorphine 12 MG / naloxone 3 MG Sublingual Film [Suboxone] |
| 40225989 | buprenorphine 2 MG / naloxone 0.5 MG Sublingual Film |
| 40225990 | buprenorphine 2 MG / naloxone 0.5 MG Sublingual Film [Suboxone] |
| 1133231 | buprenorphine 2 MG / naloxone 0.5 MG Sublingual Tablet |
| 40225991 | buprenorphine 2 MG / naloxone 0.5 MG Sublingual Tablet [Suboxone] |
| 1133229 | buprenorphine 2 MG Sublingual Tablet |
| 19102738 | buprenorphine 2 MG Sublingual Tablet [Subutex] |
| 45776275 | buprenorphine 2.1 MG / naloxone 0.3 MG Buccal Film [Bunavail] |
| 46287543 | buprenorphine 2.9 MG / naloxone 0.71 MG Sublingual Tablet |
| 46287551 | buprenorphine 2.9 MG / naloxone 0.71 MG Sublingual Tablet [Zubsolv] |
| 793568 | buprenorphine 200 MG/ML Prefilled Syringe [Sublocade] |
| 42898503 | buprenorphine 4 MG / naloxone 1 MG Sublingual Film |
| 42898504 | buprenorphine 4 MG / naloxone 1 MG Sublingual Film [Suboxone] |
| 45774518 | buprenorphine 4.2 MG / naloxone 0.7 MG Buccal Film |
| 45774520 | buprenorphine 4.2 MG / naloxone 0.7 MG Buccal Film [Bunavail] |
| 43532944 | buprenorphine 5.7 MG / naloxone 1.4 MG Sublingual Tablet |
| 43532945 | buprenorphine 5.7 MG / naloxone 1.4 MG Sublingual Tablet [Zubsolv] |
| 45774521 | buprenorphine 6.3 MG / naloxone 1 MG Buccal Film |
| 45774523 | buprenorphine 6.3 MG / naloxone 1 MG Buccal Film [Bunavail] |
| 40225993 | buprenorphine 8 MG / naloxone 2 MG Sublingual Film |
| 40225994 | buprenorphine 8 MG / naloxone 2 MG Sublingual Film [Suboxone] |
| 1133262 | buprenorphine 8 MG / naloxone 2 MG Sublingual Tablet |
| 40225995 | buprenorphine 8 MG / naloxone 2 MG Sublingual Tablet [Suboxone] |
| 1133230 | buprenorphine 8 MG Sublingual Tablet |
| 19102739 | buprenorphine 8 MG Sublingual Tablet [Subutex] |
| 45892572 | buprenorphine 8.6 MG / naloxone 2.1 MG Sublingual Tablet |
| 45892574 | buprenorphine 8.6 MG / naloxone 2.1 MG Sublingual Tablet [Zubsolv] |
| 40015181 | buprenorphine Sublingual Tablet |
| 1714319 | naltrexone |
| 1714351 | naltrexone 380 MG Injection |
| 1714372 | naltrexone 380 MG Injection [Vivitrol] |
| 44784878 | naltrexone hydrochloride 50 MG Oral Tablet |
| 44784879 | naltrexone hydrochloride 50 MG Oral Tablet [Depade] |
| 44784880 | naltrexone hydrochloride 50 MG Oral Tablet [ReVia] |
