# Supplementary material for: Real-world management of opioid use disorder in primary care 2015–2019: associations between clinical practice attributes, diagnosis, and treatment
Source: Crit Public Health. Author manuscript; Available in PMC 2026 Jul 11. (PMC13354046; doi:10.1080/09581596.2026.2676423)
Supplement: Supplemental Table 2 [file NIHMS2181851-supplement-Supplemental_Table_2.docx]

Supplemental Table 2. Distribution of race and ethnicity among primary care patients with opioid use disorder prior to imputation for race: Stratified by prescription status for medications for opioid use disorder (n=28,168)

|  | **No MOUD (N=18,753)** | **Prescribed MOUD (N=9415)** | **Total (N=28,168)** |
| --- | --- | --- | --- |
| **Race** |  |  |  |
| American Indian or Alaska Native | 124 (0.7%) | 68 (0.7%) | 192 (0.7%) |
| Asian | 71 (0.4%) | 17 (0.2%) | 88 (0.3%) |
| Black or African American | 1108 (5.9%) | 300 (3.2%) | 1408 (5.0%) |
| Multiple races | 6 (0.0%) | 3 (0.0%) | 9 (0.0%) |
| Native Hawaiian or Other Pacific Islander | 57 (0.3%) | 11 (0.1%) | 68 (0.2%) |
| Unknown race | 5911 (31.5%) | 2487 (26.4%) | 8398 (29.8%) |
| White | 11,476 (61.2%) | 6529 (69.3%) | 18,005 (63.9%) |
| **Ethnicity** |  |  |  |
| Hispanic or Latino | 1380 (7.4%) | 233 (2.5%) | 1613 (5.7%) |
| Not Hispanic or Latino | 8895 (47.4%) | 4848 (51.5%) | 13,743 (48.8%) |
| Unknown ethnicity | 8478 (45.2%) | 4334 (46.0%) | 12,812 (45.5%) |
