# Supplementary material for: Real-world management of opioid use disorder in primary care 2015–2019: associations between clinical practice attributes, diagnosis, and treatment
Source: Crit Public Health. Author manuscript; Available in PMC 2026 Jul 11. (PMC13354046; doi:10.1080/09581596.2026.2676423)
Supplement: Supplemental Table 3 [file NIHMS2181851-supplement-Supplemental_Table_3.docx]

Supplemental Table 3. Distribution of primary care practices by practice rank* and rural urban commuting area codes, n=854 primary care practices

| **RUCA** | **Practice rank** | **Frequency** | **%** | **Cumulative %** |
| --- | --- | --- | --- | --- |
| Metropolitan Area | 1^st^ to 5^th^ | 4 | 0.5 | 0.5 |
|  | 6^th^ to 15^th^ | 8 | 0.9 | 1.4 |
|  | 16^th^ to 50^th^ | 26 | 3.0 | 4.4 |
|  | 51^st^ to 100^th^ | 38 | 4.4 | 8.8 |
|  | 101^st^ to 854^th^ | 520 | 60.9 | 69.7 |
| Micropolitan Area | 6^th^ to 15^th^ | 1 | 0.1 | 69.8 |
|  | 16^th^ to 50^th^ | 5 | 0.6 | 70.4 |
|  | 51^st^ to 100^th^ | 6 | 0.7 | 71.1 |
|  | 101^st^ to 854^th^ | 130 | 15.2 | 86.3 |
| Small Town | 1^st^ to 5^th^ | 1 | 0.1 | 86.4 |
|  | 6^th^ to 15^th^ | 1 | 0.1 | 86.5 |
|  | 16^th^ to 50^th^ | 3 | 0.4 | 86.9 |
|  | 51^st^ to 100^th^ | 5 | 0.6 | 87.5 |
|  | 101^st^ to 854^th^ | 68 | 8.0 | 95.5 |
| Rural Area | 16^th^ to 50^th^ | 1 | 0.1 | 95.6 |
|  | 51^st^ to 100^th^ | 1 | 0.1 | 95.7 |
|  | 101^st^ to 854^th^ | 32 | 3.7 | 99.4 |
| Unknown | 101^st^ to 854^th^ | 4 | 0.5 | 99.9 |

* Practice rank is assigned by the total number of patients with a diagnosis of opioid use disorder during the study period 2015-2019
