# Supplementary figures and images for: Real-world management of opioid use disorder in primary care 2015–2019: associations between clinical practice attributes, diagnosis, and treatment
Source: Crit Public Health. Author manuscript; Available in PMC 2026 Jul 11. (PMC13354046; doi:10.1080/09581596.2026.2676423)

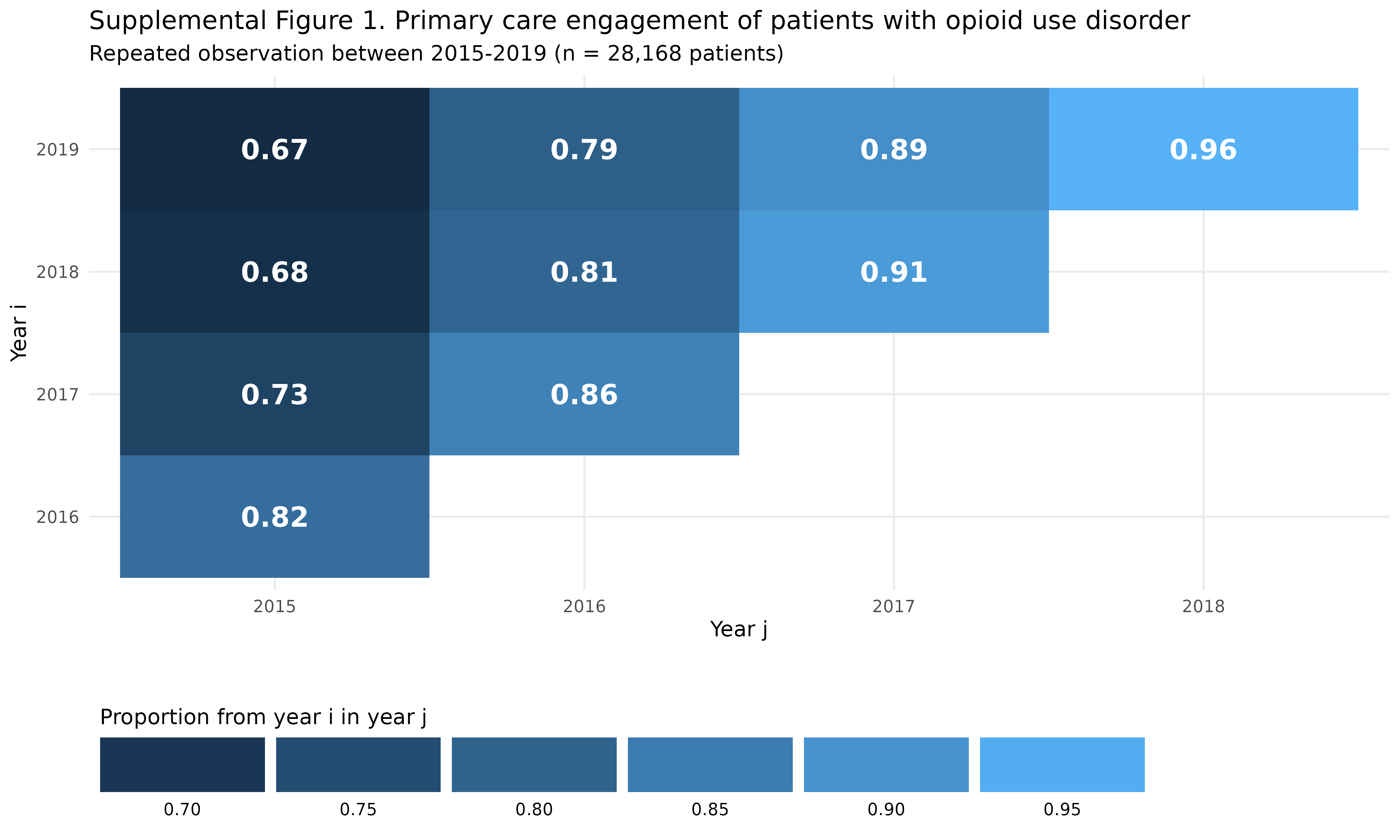

Supplement: Supplemental Figure 1 [file NIHMS2181851-supplement-Supplemental_Figure_1.png]
